# Diabetes, Prediabetes and the Survival of Nasopharyngeal Carcinoma: A Study of 5,860 Patients

**DOI:** 10.1371/journal.pone.0111073

**Published:** 2014-10-28

**Authors:** Pu-Yun OuYang, Zhen Su, Jie Tang, Xiao-Wen Lan, Yan-Ping Mao, Wuguo Deng, Fang-Yun Xie

**Affiliations:** 1 Department of Radiation Oncology, Sun Yat-sen University Cancer Center, State Key Laboratory of Oncology in South China, Collaborative Innovation Center for Cancer Medicine, Guangzhou, Guangdong, China; 2 Department of Experimental Research, Sun Yat-sen University Cancer Center, State Key Laboratory of Oncology in South China, Collaborative Innovation Center for Cancer Medicine, Guangzhou, Guangdong, China; Central South University, China

## Abstract

**Background:**

The incidence of diabetes is increasing. But the impact of diabetes and prediabetes on survival of patients with nasopharyngeal carcinoma (NPC) has received little evaluation.

**Methods:**

In a cohort of 5,860 patients, we compared the disease specific survival (DSS), locoregional relapse-free survival (LRFS) and distant metastasis-free survival (DMFS) of patients with diabetes, prediabetes and normoglycemia defined by pretreatment fasting plasma glucose (FPG) using Kaplan–Meier method, log-rank test and Cox proportional hazards model.

**Results:**

Comparing to normoglycemic patients, the diabetic and the prediabetic were generally older, fatter, had hypertension, heart diseases and hyperlipaemia and usually received radiotherapy alone. But both the diabetic and the prediabetic had similar DSS, LRFS and DMFS to normoglycemic patients, even adjusting for such important factors as age, gender, smoking, drinking, hypertension, heart diseases, body mass index, hyperlipaemia, titer of VCA-IgA and EA-IgA, pathology, T-stage, N-stage, chemotherapy and radiotherapy (*P*>0.05 for all). Additionally, the findings remained unchanged in sensitivity analysis by excluding patients with known diabetes history and in subgroups of the various factors.

**Conclusions:**

The diabetic and prediabetic NPC patients had similar survival to normoglycemic NPC patients. These data, in the largest reported cohort, are the first to evaluate the association between diabetes, prediabetes and the survival in NPC. The findings are relevant to patient management and provided evidence of the effect on this disease exerted by comorbidities.

## Introduction

The incidence of diabetes is increasing worldwide. Epidemiologic evidence suggests that people with diabetes are at an increased risk of cancers of liver, biliary tract, pancreatic, colorectal, as well as leukemia and melanoma [1]–[3]. Importantly, clinical studies observed a significantly poorer survival in several kinds of cancer patients with elevated blood glucose levels than those with normoglycemia, including extranodal natural killer (NK)/T-cell lymphoma (nasal type) [4], lung cancer [5], pancreatic cancer [6], breast cancer [7]–[9], acute lymphocytic leukemia [10] or colorectal cancer [11], [12].

However, no studies found significant association between diabetes and a higher risk of head and neck cancer [13], [14]. And Stott-Miller even observed weak inverse associations between type 2 diabetes and head and neck squamous cell cancer (HNSCC) [13], which was quite similar to the relation of diabetes with a lower risk of larynx cancer in the study by Atchison et al [3]. Additionally, nasopharyngeal carcinoma (NPC) is a non-lymphomatous, squamous-cell carcinoma that occurs in the epithelial lining of the nasopharynx. Of particular importance, it has distinct epidemiology, etiology [15], pathologic characteristics, clinical manifestation and treatment modes [16] compared to other cancers, including other types of head and neck cancer. Therefore, the finding that other types of cancer patients with diabetes had a lower survival than those without diabetes cannot be directly applied to the patients with NPC. To our best knowledge, only one study had reported the association between diabetes and the survival of NPC patients [17]. Unfortunately, only 37 patients with diabetes at diagnosis of NPC were enrolled into that study, and the influence of obesity, smoking, hypertension, heart diseases and hyperlipaemia were not taken into account.

In this largest study, with adjustment for various important covariates, we would provide convincing evidence of the association between diabetes, prediabetes defined by fasting plasma glucose (FPG) and the survival of NPC patients.

## Materials and Methods

### Patients

The study was reviewed and approved by the Human Ethics Approval Committee at Sun Yat-sen University Cancer Center. As a retrospective analysis of routine data, we therefore requested and were granted a waiver of individual informed consent from the ethics committee. Between January 2005 and December 2010, 6034 newly diagnosed, biopsy-proven, non-metastatic and hospitalized NPC patients who were at the age of 20 or>20 years were potentially eligible for this study. After excluding cases with missing data, we eventually enrolled 5860 patients who had complete pretreatment evaluation including history and physical examination, haematology and biochemistry profiles, fiberoptic nasopharyngoscopy with biopsy, magnetic resonance imaging (MRI) of the nasopharynx and neck, chest radiography, abdominal sonography and Technetium-99m-methylene diphosphonate (Tc-99-MDP) whole-body bone scan. The following pretreatment data were anonymously extracted and analyzed, including age, gender, smoking status, drinking status, hypertension history, heart diseases history, diabetes history, FPG, body mass index (BMI), total cholesterol (CHO), triglycerides (TG), high density lipoprotein cholesterol (HDL-C), low density lipoprotein cholesterol (LDL-C), titer of immunoglobulin A against viral capsid antigen (VCA-IgA) and early antigen (EA-IgA) and histological type.

All the included patients were restaged according to the seventh edition of the UICC/AJCC Staging System for NPC [18]. And all were treated by definitive intensity-modulated radiotherapy (IMRT) or conventional radiotherapy (CRT) with or without chemotherapy; further details of the radiation techniques had been described previously [19]. Institutional guidelines recommended no chemotherapy for patients in early stage, and induction, concurrent and adjuvant chemotherapy or combined treatment for those in locoregionally advanced stage. Induction or adjuvant chemotherapy consisted of cisplatin with 5-fluorouracil, cisplatin with taxane or triplet of cisplatin and 5-fluorouracil plus taxane every 3 weeks for two to three cycles. Concurrent chemotherapy consisted of cisplatin given on weeks 1, 4 and 7 of radiotherapy or cisplatin given weekly. Deviation from the institutional guidelines was result from organ dysfunction, treatment intolerance and/or patient refusal.

Patients were examined every 3–6 months during the first 3 years, with follow-up examinations every 6–12 months thereafter or until death. The assessment included history and physical examination and a series of conventional examination equipment at each follow-up visit, to detect the possible relapse or distant metastasis. Local relapses were confirmed by biopsy, MRI scan, or both. Regional relapses were diagnosed by clinical examination and an MRI scan of the neck and, in doubtful cases, by fine needle aspiration of the lymph nodes. Distant metastases were diagnosed by clinical symptoms, physical examinations, and imaging methods including chest radiography, bones scan, MRI, and abdominal sonography. Patients with relapse, distant metastasis or in persistent disease were delivered with salvage treatment including reirradiation, chemotherapy and surgery. Those patients without recent examination tests in the medical records were followed up by telephone call.

### Diabetes and prediabetes assessment

According to the 2014 diagnosis and classification of diabetes mellitus by American Diabetes Association (ADA) [20], patients were classified into the normoglycemic (FPG <5.6 mmol/L), the prediabetic (FPG 5.6–6.9 mmol/L) and the diabetic (FPG ≥7.0 mmol/L) group based on FPG only. Patients with known diabetes at diagnosis were classified into the diabetic group and were excluded in sensitivity analysis.

### End points

The primary end point was disease specific survival (DSS), defined as the time from treatment to death resulting from NPC or treatment complications [21]. Secondary end points were locoregional relapse-free survival (LRFS) and distant metastasis-free survival (DMFS), defined as the time from treatment to the first locoregional relapse and distant metastasis, respectively.

### Statistical analysis

Statistical analyses were performed using IBM SPSS Statistics version 20.0. Clinical parameters, including CHO, TG, HDL-C and LDL-C, were stratified into normal and abnormal group. Age and titer of VCA-IgA and EA-IgA were classified according to the criteria adopted in the previous studies [22], [23]. Comparisons of categorical characteristics were performed using χ^2^ statistic. Univariate stratified survival analyses were performed using Kaplan–Meier methods and log-rank test [24]. Multivariate analyses for hazard ratios (HRs) and 95% confidence intervals (CIs) were performed using the Cox proportional hazards model [25] with forward selection method for important covariates such as gender, smoking and BMI, and enter method for FPG. Two-sided *P*-values <0.05 were considered to be significant.

## Results

### Patients

The median follow-up duration (from the first day of therapy) was 55.6 months (range, 3.1–119.2 months), with 612 (10.4%) cases of lost-to-follow up. There were 569 (9.7%) cases of locoregional relapse, 762 (13.0%) cases of distant metastasis and 889 (15.2%) cases of disease-cause death, respectively. The 5-year survival rates were as follows: DSS 84.9%, LRFS 89.2% and DMFS 86.0%.

The clinicopathologic characteristics of the 5860 patients were shown in Table 1. Of the 121 patients who had known diabetes at diagnosis, 17 patients had a FPG level <5.6 mmol/L and 44 patients <7.0 mmol/L. Drinking, HDL-C level, titer of VCA-IgA and EA-IgA, histological type, T-stage, N-stage, clinical stage and radiotherapy did not significantly differ for group of the diabetic versus the normoglycemic or the prediabetic versus the normoglycemic. Comparing to normoglycemic patients, the diabetic and the prediabetic were generally older, fatter, had hypertension, heart diseases and higher levels of CHO, TG and LDL-C and usually received radiotherapy alone. In the diabetic group, we observed a significantly higher proportion of smoker.

**Table 1:** Clinicopathologic characteristics of 5860 patients with nasopharyngeal carcinoma.

Characteristics	Normoglycemia	Diabetes	Prediabetes	*P1*	*P2*
	No. (%)	No. (%)	No. (%)		
**Total**	3949	345	1566		
**Age**				**<0.001**	**<0.001**
20–30	254 (6.4)	3 (0.9)	26 (1.7)		
30–40	1138 (28.8)	26 (7.5)	290 (18.5)		
40–50	1286 (32.6)	104 (30.1)	553 (35.3)		
50–60	875 (22.2)	104 (30.1)	445 (28.4)		
≥60	396 (10.0)	108 (31.3)	252 (16.1)		
**Gender**				**0.072**	**0.133**
Male	2916 (73.8)	270 (78.3)	1187 (75.8)		
Female	1033 (26.2)	75 (21.7)	379 (24.2)		
**Smoking**				**0.004**	**0.862**
Yes	1677 (42.5)	174 (50.4)	661 (42.2)		
No	2272 (57.5)	171 (49.6)	905 (57.8)		
**Drinking**				**0.495**	**0.107**
Yes	477 (12.1)	46 (13.3)	165 (10.5)		
No	3472 (87.9)	299 (86.7)	1401 (89.5)		
**Hypertension**				**<0.001**	**0.012**
Yes	169 (4.3)	67 (19.4)	92 (5.9)		
No	3780 (95.7)	278 (80.6)	1474 (94.1)		
**Heart disease**				**<0.001**	**<0.001**
Yes	25 (0.6)	46 (13.3)	28 (1.8)		
No	3924 (99.4)	299 (86.7)	1538 (98.2)		
**BMI (kg/m^2^)** §				**<0.001**	**<0.001**
<18.5	346 (8.8)	11 (3.2)	85 (5.4)		
18.5–22.9	1819 (46.1)	85 (24.6)	562 (35.9)		
22.9–27.5	1505 (38.1)	194 (56.2)	744 (47.5)		
≥ 27.5	279 (7.1)	55 (15.9)	175 (11.2)		
**CHO (mmol/L)** ¶				**<0.001**	**<0.001**
≤6.47	3629 (91.9)	296 (85.8)	1367 (87.3)		
>6.47	320 (8.1)	49 (14.2)	199 (12.7)		
**TG (mmol/L)** ¶				**<0.001**	**0.003**
≤1.7	2826 (71.6)	190 (55.1)	1058 (67.6)		
>1.7	1123 (28.4)	155 (44.9)	508 (32.4)		
**HDL-C (mmol/L)** ¶				**0.954**	**0.402**
≥0.78	3848 (97.4)	336 (97.4)	1532 (97.8)		
<0.78	101 (2.6)	9 (2.6)	34 (2.2)		
**LDL-C (mmol/L)** ¶				**0.001**	**<0.001**
≤3.4	2416 (61.2)	179 (51.9)	839 (53.6)		
>3.4	1533 (38.8)	166 (48.1)	727 (46.4)		
**VCA-IgA #**				**0.085**	**0.334**
≤80	988 (25.0)	73 (21.2)	418 (26.7)		
80–320	2024 (51.3)	198 (57.4)	771 (49.2)		
>320	937 (23.7)	74 (21.4)	377 (24.1)		
**EA-IgA #**				**0.076**	**0.293**
≤10	1755 (44.4)	136 (39.4)	673 (43.0)		
10–40	1260 (31.9)	130 (37.7)	534 (34.1)		
>40	934 (23.7)	79 (22.9)	359 (22.9)		
**Histological type** *				**0.598**	**0.097**
WHO I+II	279 (7.1)	27 (7.8)	131 (8.4)		
WHO III	3670 (92.9)	318 (92.2)	1435 (91.6)		
**T-stage**				**0.804**	**0.070**
T1+T2	1450	129	616		
T3+T4	2499	216	950		
**N-stage**				**0.246**	**0.124**
N0+N1	3017	254	1205		
N2+N3	932	91	333		
**Clinical stage**				**0.139**	**0.221**
I	223 (5.6)	12 (3.5)	102 (6.5)		
II	918 (23.2)	81 (23.5)	395 (25.2)		
III	1569 (39.7)	146 (42.3)	618 (39.5)		
IVa	1047 (26.5)	82 (23.8)	382 (24.4)		
IVb	192 (4.9)	24 (7.0)	69 (4.4)		
**Chemotherapy**				**0.003**	**0.005**
No	725 (18.4)	86 (24.9)	339 (21.6)		
Yes	3224 (81.6)	259 (75.1)	1227 (78.4)		
**Radiotherapy**				**0.632**	**0.084**
IMRT	1161 (29.4)	109 (31.6)	456 (29.1)		
3DCRT	59 (1.5)	4 (1.2)	37 (2.4)		
2DCRT	2729 (69.1)	232 (67.2)	1073 (68.5)		

Note: BMI  =  body mass index, CHO  =  total cholesterol, TG  =  triglycerides, HDL-C  =  high density lipoprotein cholesterol, LDL-C  =  low density lipoprotein cholesterol, VCA  =  viral capsid antigen, EA  =  early antigen, IgA  =  immunoglobulin A, IMRT  =  intensity-modulated radiotherapy, 3DCRT  =  three-dimensional conformal radiotherapy, 2DCRT  =  two-dimensional conventional radiotherapy.

*P1* – diabetes *vs* normoglycemia; *P2* – prediabetes *vs* normoglycemia.

§ According to the World Health Organization classifications for Asian populations.

¶ Stratified into normal and abnormal group.

# In accordance with the criteria adopted in the previous study.

*Based on the criteria of WHO histological type (1991): I - Squamous-cell carcinomas, II - Differentiated non-keratinising carcinoma, III - Undifferentiated non-keratinising carcinoma.

### Diabetes, prediabetes and survival

**Figure 1:**
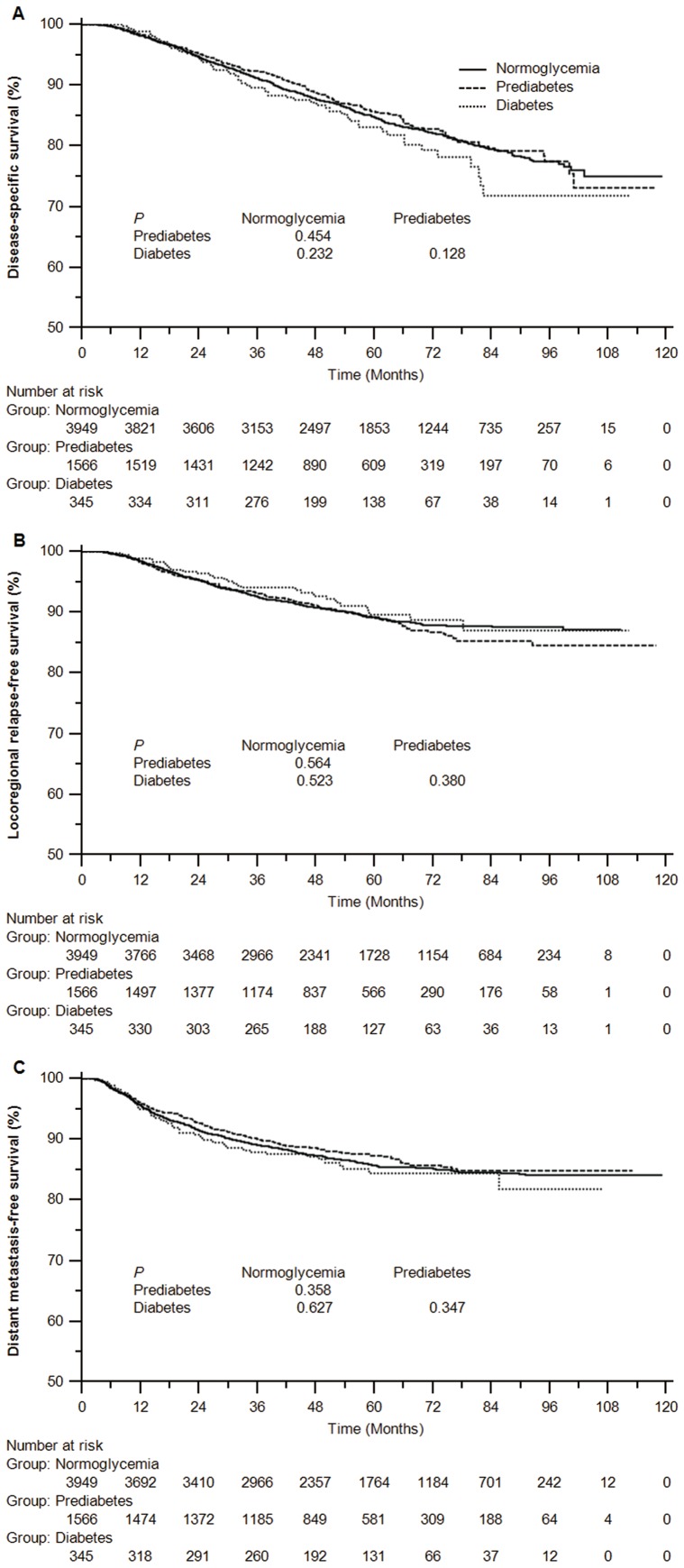
Kaplan-Meier curves of disease specific survival (A), locoregional relapse-free survival (B) and distant metastasis-free survival (C) for patients with normoglycemia, prediabetes and diabetes mellitus defined by fasting plasma glucose.

In contrast with normoglycemic patients, Kaplan-Meier curves displayed the non-significant differences of DSS, LRFS and DMFS rates for patients with diabetes or prediabetes. (Figure 1)

Since diabetes or prediabetes was usually accompanied with age, obesity, smoking, hypertension, heart diseases and hyperlipaemia, the actual survival differences between diabetic, prediabetic and normoglycemic NPC patients cannot be disclosed exactly without excluding the influence of these covariates. However, after adjusting for age, gender, smoking, drinking, hypertension, heart diseases, BMI, levels of CHO, TG, HDL-C and LDL-C, titer of VCA-IgA and EA-IgA, histological type, T-stage, N-stage, chemotherapy and radiotherapy, we still found no significant differences of DSS, LRFS and DMFS when comparing patients with diabetes to those with normoglycemia (*P* = 0.894 for DSS, *P* = 0.351 for LRFS and *P* = 0.530 for DMFS) and comparing patients with prediabetes to those with normoglycemia (*P* = 0.335 for DSS, *P* = 0.613 for LRFS and *P* = 0.671 for DMFS). (Table 2)

**Table 2:** Multivariate analysis for disease specific survival (DSS), locoregional relapse-free survival (LRFS) and distant metastasis-free survival (DMFS) *.

Factor	DSS			LRFS			DMFS		
	HR	95% CI	*P*	HR	95% CI	*P*	HR	95% CI	*P*
Normoglycemia	1.00			1.00			1.00		
Diabetes	0.98	0.75–1.29	0.894	0.83	0.57–1.22	0.351	1.10	0.81–1.49	0.530
Prediabetes	0.93	0.79–1.08	0.335	1.05	0.87–1.27	0.613	0.96	0.82–1.14	0.671
Gender	0.64	0.54–0.76	<0.001	0.65	0.53–0.80	<0.001	0.64	0.54–0.77	<0.001
Age	1.47	1.38–1.57	<0.001	1.09	1.01–1.18	0.030	1.09	1.02–1.16	0.017
T-stage	1.53	1.42–1.64	<0.001	1.28	1.18–1.39	<0.001	1.45	1.35–1.57	<0.001
N-stage	1.61	1.51–1.72	<0.001	1.27	1.16–1.39	<0.001	1.72	1.61–1.85	<0.001
BMI	0.81	0.74–0.88	<0.001	NS			0.84	0.76–0.92	<0.001

NOTE: HR  =  hazard ratio, CI  =  confidence interval, BMI  =  body mass index.

*Adjusting for age, gender, smoking, drinking, hypertension, heart diseases, BMI, levels of total cholesterol, triglycerides, high density lipoprotein cholesterol and low density lipoprotein cholesterol, titer of VCA-IgA and EA-IgA, histological type, T-stage, N-stage, chemotherapy and radiotherapy with forward selection method.

To fully eliminate the effect of the discrepancies as a result of the normal or prediabetic FPG level for the 121 patients with known diabetes history, we did sensitivity analysis by excluding them. Consequently, the above results remained unchanged, as shown in Table S1.

In addition, we performed second analyses stratified by several important subgroups. (Table 3) Resultantly, multivariate analyses indicated that neither diabetes nor prediabetes was significantly associated with DSS in subgroups of age (≤45 and>45 y), gender, smoking, drinking, hypertension, heart diseases, BMI (<25 and ≥25 kg/m^2^), CHO, TG, HDL-C, LDL-C, T-stage (T1+T2 and T3+T4), N-stage (N0+N1 and N2+N3), clinical stage (I+II and III+IV), chemotherapy and radiotherapy (2DCRT and IMRT + 3DCRT).

**Table 3:** Subgroup analysis of disease specific survival by patients' characteristics*.

Factor	Diabetes			Prediabetes		
	HR	95% CI	*P*	HR	95% CI	*P*
**Age (year)** §						
≤45	1.51	0.86–2.64	0.155	1.06	0.80–1.40	0.691
>45	0.98	0.72–1.33	0.877	0.88	0.73–1.06	0.184
**Gender**						
Male	1.13	0.85–1.51	0.388	0.90	0.75–1.07	0.215
Female	0.83	0.57–1.22	0.351	1.07	0.75–1.54	0.701
**Smoking**						
Yes	0.98	0.69–1.39	0.893	0.93	0.75–1.16	0.535
No	0.96	−.62–1.48	0.835	0.90	0.71–1.13	0.364
**Drinking**						
Yes	0.97	0.49–1.94	0.934	1.19	0.81–1.76	0.377
No	1.00	0.74–1.34	0.986	0.89	0.75–1.06	0.180
**Hypertension**						
Yes	0.73	0.34–1.58	0.428	0.93	0.48–1.80	0.824
No	1.02	0.76–1.38	0.872	0.94	0.79–1.10	0.415
**Heart diseases**						
Yes	0.49	0.20–1.20	0.118	0.41	0.14–1.22	0.109
No	0.99	0.74–1.33	0.937	0.94	0.80–1.10	0.422
**BMI (kg/m^2^)** §						
<25	0.91	0.64–1.29	0.600	0.87	0.72–1.05	0.152
≥25	1.03	0.66–1.62	0.884	0.98	0.74–1.32	0.914
**CHO (mmol/L)**						
≤6.47	0.97	0.72–1.30	0.833	0.93	.79–1.10	0.394
>6.47	0.92	0.43–2.00	0.837	0.78	0.48–1.26	0.309
**TG (mmol/L)**						
≤1.7	1.23	0.88–1.72	0.230	0.96	0.79–1.16	0.669
>1.7	0.66	0.41–1.06	0.084	0.87	0.65–1.15	0.324
**HDL-C mmol/L)**						
≥0.78	0.93	0.70–1.23	0.595	0.91	0.78–1.07	0.912
<0.78	1.39	0.44–4.36	0.577	1.30	0.59–2.83	0.518
**LDL-C (mmol/L)**						
≤3.4	0.92	0.62–1.36	0.666	1.03	0.84–1.28	0.759
>3.4	1.03	0.71–1.51	0.873	0.83	0.65–1.05	0.114
**T-stage**						
T1+T2	1.10	0.65–1.84	0.730	0.80	0.58–1.13	0.203
T3+T4	0.89	0.64–1.22	0.464	0.95	0.79–1.13	0.534
**N-stage**						
N0+N1	0.72	0.49–1.06	0.093	0.89	0.73–1.09	0.269
N2+N3	1.08	0.72–1.63	0.699	0.91	0.70–1.17	0.465
**Clinical stage**						
I+II	1.07	0.55–2.09	0.846	0.70	0.46–1.08	0.106
III+ IV	0.89	0.66–1.20	0.440	0.95	0.80–1.12	0.512
**Chemotherapy**						
Yes	1.04	0.77–1.42	0.784	0.98	0.83–1.16	0.811
No	0.63	0.34–1.14	0.124	0.68	0.45–1.02	0.060
**Radiotherapy**						
2DCRT	1.11	0.82–1.51	0.498	0.90	0.75–1.08	0.256
IMRT + 3DCRT	0.68	0.37–1.28	0.231	1.03	0.76–1.40	0.860

NOTE: HR  =  hazard ratio, CI  =  confidence interval, BMI  =  body mass index, CHO  =  total cholesterol, TG  =  triglycerides, HDL-C  =  high density lipoprotein cholesterol, LDL-C  =  low density lipoprotein cholesterol, VCA  =  viral capsid antigen, EA  =  early antigen, IgA  =  immunoglobulin A, 2DCRT  =  two-dimensional conventional radiotherapy, IMRT  =  intensity-modulated radiotherapy, 3DCRT  =  three-dimensional conformal radiotherapy.

*Adjusting for age, gender, smoking, drinking, hypertension, heart diseases, BMI, levels of total cholesterol, triglycerides, high density lipoprotein cholesterol and low density lipoprotein cholesterol, titer of VCA-IgA and EA-IgA, histological type, T-stage, N-stage, chemotherapy and radiotherapy.

§ According to the stratification criteria for the risk factor of age and BMI mentioned in the 2014 diagnosis and classification of diabetes mellitus by American Diabetes Association (ADA).

## Discussion

Based on 5860 patients and thoroughly adjusting for the influence of age, obesity, smoking, drinking, hypertension, heart diseases, hyperlipaemia, tumor stage and treatment modality, our study concluded that the diabetic and prediabetic NPC patients had similar survival to the normoglycemic NPC patients.

In contrast to our present study, Liu et al [17] detected a lower disease-free survival in patients with diabetes (n = 37) than those without diabetes (n = 897); nevertheless, this study did not account for all the various potential confounders, such as obesity, smoking, hypertension, heart diseases and hyperlipaemia. Similar studies also found the significant association between hyperglycemia and the survival of patients with extranodal natural killer (NK)/T-cell lymphoma (nasal type) [4] or acute lymphocytic leukemia [10], and between DM and the survival of patients with lung cancer [5], pancreatic cancer [6], breast cancer [7], [8] or colorectal cancer [11], [12]. But this is hardly convincing as the small sample size of these studies [4]–[6], [10] is very likely to cause the skewed results.

Actually, Zhou et al [26] recruited 26,460 men and 18,195 women aged 25–90 years from 17 European population-based or occupational cohorts and found that diabetes was not significantly associated with the mortality of male patients with cancers of pancreas, bronchus/lung, prostate and kidney/bladder, or the mortality of female patients with cancers of stomach or colon – rectum, bronchus/lung, breast and kidney/bladder. Also, Höfner et al [27] enrolled 1140 patients with localized renal cell carcinoma and revealed that type 2 diabetes at the time of surgery had no significant impact on cancer-specific and recurrence-free survival. In the study by Kiderlen et al [8], relapse-free period was better in elderly breast cancer patients with diabetes compared with patients without diabetes if taking competing mortality into account; patients with diabetes without other comorbidity had a similar overall survival as patients without any comorbidity. Additionally, the ORIGIN trial found no evidence for increased cancer incidence or mortality in patients with impaired glucose metabolism or early type 2 diabetes [28]. Overall, despite of absence of straight evidence regarding the impact of FPG on survival of other types of head and neck cancer, our findings of neutral impact in NPC patients were not unreasonable. Finally, the non-significant association between diabetes and risk of head and neck cancer from the prior pooled analysis [13] and meta-analysis [14], along with the inverse relationship between diabetes and development of larynx cancer in another cohort study [3], at least indirectly suggested no impact of FPG on the survival of NPC.

Previous research showed that cancer patients with diabetes may have increased tumor cell proliferation and metastatic capacity as a consequence of the high insulin or increased free insulin-like growth factor (IGF-1) levels in hyperinsulinemic states [29]. But this was denied by a recent study [30], in which incident insulin users, exposure to insulin and glargine insulin in particular was not associated with any deleterious effect on overall and site specific cancer mortality of lung, colorectal, female genital, liver and urinary tract cancer. What is more, Margel et al [31] discovered that increased cumulative duration of metformin exposure after prostate cancer diagnosis was associated with decreases in both all-cause and cancer-specific mortality among diabetic men. But this similar protective effect from metformin exposure was not supported in NPC patients with diabetes in our study.

Certainly, the pooled analysis by Stott-Miller et al [13] showed a modest association between diabetes and the incidence of head and neck cancer among never smokers. And Atchison et al [3] assumed that smoking and BMI were two important factors potentially contributed to the inverse relationship between diabetes and development of larynx cancer. As observed in our study, a higher percentage of diabetic patients were indeed smokers and overweight or obese. Additionally, according to recent studies, NPC patients with smoking history had poorer survival [32] whereas those with higher BMI had favorable survival [33]. Therefore, the contradictory effect of smoking and BMI maybe just right principally confounded the impact of FPG on survival of NPC. However, in the stratum of patients who had normal BMI and never smoked (n = 1461), multivariate analysis showed that both diabetic and prediabetic patients had similar DSS, LRFS and DMFS rates to euglycemic patients (*P* = 0.298, *P* = 0.613 and *P* = 0.433 for DSS; *P* = 0.554, *P* = 0.315 and *P* = 0.693 for LRFS; *P* = 0.434, *P* = 0.747 and *P* = 0.458 for DMFS, respectively).

Considering the influence of mortality from such hyperglycemia-related complications as hypertension, heart diseases and various hyperlipaemia, we set DSS as the primary endpoint, adjusted for these covariates and conducted subgroup analyses; finally, diabetes or prediabetes still had null influence to NPC survival. Moreover, there were no significant differences with respect to the distribution of tumor stage and radiotherapy, and diabetes or prediabetes remained to be irrelevant to the survival in these subgroups. Particularly, patients with diabetes or prediabetes usually received radiotherapy alone with a higher percentage than that of patients with normoglycemia. But this rarely affected the DSS of the diabetic or prediabetic subgroups.

To our knowledge, this is the largest and most detailed study to evaluate the relation between diabetes, prediabetes before treatment and the survival of NPC patients. Clinicopathologic and survival data were verified by review of individual patient records. Our findings were derived from complete adjustment and particular stratification of various important covariates. The conclusions are relevant to patient management and provided evidence of the effect on the disease of NPC exerted by comorbidities. Indeed, albeit that FPG is the primary routine test in clinic, further study with data on standard 2-hour oral glucose tolerance test (OGTT) and hemoglobin A1c (HbA1c) is warranted. Apart from that, the effect of glycemic control during radiotherapy and chemotherapy on the survival is essential to be studied.

## Supporting Information

Table S1 Sensitivity analysis by excluding the 121 patients with known diabetes history *.
(DOCX)
