# Supplementary material for: Diabetes, Prediabetes and the Survival of Nasopharyngeal Carcinoma: A Study of 5,860 Patients
Source: PLoS One. 2014 Oct 28;9(10):e111073. doi: 10.1371/journal.pone.0111073 (PMC4211733; doi:10.1371/journal.pone.0111073)
Supplement: Table S1 — Sensitivity analysis by excluding the 121 patients with known diabetes history *. (DOCX) [file pone.0111073.s001.docx]

**Table S1.** Sensitivity analysis by excluding the 121 patients with known diabetes history *

|  |  | DSS |  |  | LRFS |  |  | DMFS |  |
| --- | --- | --- | --- | --- | --- | --- | --- | --- | --- |
|  | HR | 95% CI | *P* | HR | 95% CI | *P* | HR | 95% CI | *P* |
| Normoglycemia | 1.00 |  |  | 1.00 |  |  | 1.00 |  |  |
| Diabetes | 1.03 | 0.74-1.42 | 0.866 | 0.79 | 0.49-1.28 | 0.342 | 0.94 | 0.63-1.39 | 0.936 |
| Prediabetes | 0.93 | 0.80-1.09 | 0.393 | 1.05 | 0.87-1.27 | 0.606 | 0.96 | 0.81-1.14 | 0.667 |
| Gender | 0.68 | 0.57-0.82 | <0.001 | 0.67 | 0.54-0.82 | <0.001 | 0.65 | 0.54-0.79 | <0.001 |
| Age | 1.46 | 1.37-1.56 | <0.001 | 1.09 | 1.01-1.18 | 0.034 | 1.08 | 1.01-1.16 | 0.02 |
| T-stage | 1.52 | 1.41-1.64 | <0.001 | 1.29 | 1.18-1.40 | <0.001 | 1.45 | 1.34-1.57 | <0.001 |
| N-stage | 1.59 | 1.49-1.70 | <0.001 | 1.25 | 1.14-1.38 | <0.001 | 1.71 | 1.60-1.84 | <0.001 |
| BMI | 0.81 | 0.74-0.89 | <0.001 | NS |  |  | 0.84 | 0.76-0.93 | <0.001 |

NOTE: HR = hazard ratio, CI = confidence interval, BMI = body mass index, NS = not significant

* Adjusting for age, gender, smoking, drinking, hypertension, heart diseases, BMI, levels of total cholesterol, triglycerides, high density lipoprotein cholesterol and low density lipoprotein cholesterol, titer of VCA-IgA and EA-IgA, histological type, T-stage, N-stage, chemotherapy and radiotherapy with forward selection method.
